# Simplified Method for Removing Direct Oral Anticoagulant Interference in Mechanical Coagulation Test Systems—A Proof of Concept

**DOI:** 10.3390/jcm13041042

**Published:** 2024-02-12

**Authors:** Thomas Exner, Manita Dangol, Emmanuel J. Favaloro

**Affiliations:** 1 Haematex Research Pty Ltd., Sydney, NSW 2077, Australia; tom@haematex.com (T.E.); manita@haematex.com (M.D.); 2 Haematology Department, Institute of Clinical Pathology and Medical Research (ICPMR), Sydney Centres for Thrombosis and Haemostasis, Westmead Hospital, Sydney, NSW 2145, Australia; 3 School of Dentistry and Medical Sciences, Faculty of Science and Health, Charles Sturt University, Wagga, NSW 2650, Australia; 4 School of Medical Sciences, Faculty of Medicine and Health, University of Sydney, Westmead Hospital, Westmead, NSW 2145, Australia

**Keywords:** DOACs, DOAC Stop, activated charcoal, clotting tests, mechanical clot detection

## Abstract

Background: Direct oral anticoagulants (DOACs) cause unwanted interference in various hemostasis assays, including lupus anticoagulant (LA) testing, where false positive and false negative identification may occur. DOAC Stop (DS) is an activated charcoal (AC) product used to specifically and effectively adsorb DOACs from test plasma. This process normally requires plasma treatment, centrifugation and plasma separation prior to tests, but inexperienced operators may also inadvertently transfer residual AC particles, thereby potentially adversely affecting clot detection. Methods: We hypothesized that residual DS might not be problematic for mechanical clot detection. We therefore investigated the potential impact of DS and a new DS liquid (DS-L) product on clotting tests using a mechanical clot detection system. Varying concentrations of DS were added to normal and abnormal plasmas with and without DOAC presence. Clotting tests including PT, APTT and dRVVT were performed directly in the analyzer without plasma/DS centrifugation. Results: DS up to double the recommended treatment level had only minor effects on all test results, despite completely obscuring visibility in the plasma/reagent mix. This confirms that the centrifugation step may be able to be omitted when using mechanical detection systems. Conclusions: Should DS carryover into treated plasmas occur, this should not cause issues with testing performed on mechanical clot-sensing devices. Moreover, we hypothesize that DS can be used directly in these systems, without the need for centrifugation, thereby simplifying its many potential applications.

## 1. Introduction

Direct oral anticoagulants (DOACs) are being increasingly deployed and are replacing classical anticoagulant approaches such as warfarin, or other vitamin K antagonists (VKAs) [1,2]. These drugs are used to treat or prevent thrombosis in a wide range of conditions, including atrial fibrillation and deep vein thrombosis (DVT). In other prothrombotic conditions, such as antiphospholipid syndrome (APS), the classical vitamin K antagonists (VKAs), including warfarin, may be preferred over DOACs [3]. Irrespective, DOACs cause interference in a wide range of hemostasis assays, especially coagulation-based assays, where they will inevitably prolong clot times [4,5,6,7,8,9,10,11]. Because of their ubiquitous presence in society, a high proportion of patients undertaking hemostasis testing will be on a DOAC when samples are collected [4,5,6,7,8,9,10,11]. In particular, a high proportion of patients being assessed for lupus anticoagulant (LA) will be on a DOAC at the time of testing [4]. This is since LA is a marker of APS, and APS is prothrombotic, and many of these patients will be on lifelong anticoagulation therapy. DOACs have variable effects on LA tests [4,5,6,7,8]. All LA tests will be prolonged when DOACs are present, but they will be variably prolonged [5,6,7,8]. LA testing comprises panels of tests, where a screening test is performed first (inevitably prolonged in the presence of DOACs), and then a confirmation test (plus mixing studies) is performed on samples giving prolonged screening test times [8]. Thus, all DOAC containing samples will need the three tests performed (screening, confirmation, and mixing). Moreover, DOACs affect screening and confirmation tests differently, so that prolongation may be greater in one than the other. This affects the calculation of the screen/confirm ratios used to identify or exclude LA, and thus DOACs may lead to either false positive or false negative LA findings [4]. These events may lead to inappropriate APS diagnosis/exclusion and incorrect patient management. In brief, false identification of LA may lead to a false diagnosis of APS, and application of anticoagulation therapy with potential (unnecessary) bleeding risk, whereas false exclusion of LA may lead to a missed diagnosis of APS and withholding of anticoagulation therapy with potential (unnecessary) thrombosis risk. The effects of DOACs on other hemostasis tests may alternatively cause misdiagnosis of other hemostatic diseases, depending on the effects and the tests performed [4,5,6,7].

The interference caused by DOACs can be effectively removed using DOAC neutralizers [4,9,10]. At the time of writing, there are three such products available commercially: DOAC Stop, DOAC Remove and DOAC Filter [4]. These DOAC neutralizers are not included in current test reagents and therefore require separate pre-treatment of the plasma sample and subsequent removal of the neutralizer (typically an activated carbon [AC] product) by centrifugation. DOAC Stop (DS) represents the first commercial DOAC neutralizer, reported in the literature in 2018 [11], and specifically developed for this use. DS has since been found to be very useful for extracting DOACs and similar compounds from plasma samples for subsequent use in a variety of assays [4,9,10,11,12,13,14,15,16,17,18,19,20,21,22]. DS has little or no apparent effect on clotting factors or proteins involved in the clotting mechanism [11], except perhaps for a minor reduction in tissue factor pathway inhibitor [21]. However, this has little effect on conventional clotting tests, such as those used for investigation of LA. The main tests used for this purpose are the APTT (activated partial thromboplastin time) and the dRVVT (dilute Russell viper venom time) [4]. Additional tests, especially the PT (prothrombin time) may be useful in helping to differentiate true LA from anticoagulant effects such as those caused by DOACs [4,23]. DOAC Remove was first reported in the literature in 2019 [24], and DOAC Filter in 2020 [25].

DS is normally added as a minitablet containing 5 mg of DS-AC to 1 mL of test plasma, dispersed for 5–10 min and then sedimented by rapid centrifugation [11]. The supernatant plasma, which is then free of DOACs, can then be safely used for essentially all hemostasis tests, including PT, APTT and dRVVT [4,9,10,11]. Because of the need to centrifuge the DS-AC out of the solution, since it interferes with clot detection using optical detection systems, this process adds roughly 10 min of a technician’s time per test sample. In the Westmead laboratory, upwards of 50 samples are tested for LA in a single day. If there was a need to pretreat every sample with DS, this would add nearly 8 h to the daily workload. Thankfully, current recommendations are to only pre-treat samples likely to contain DOACs [8,10,23]. Thus, the main residual concern is that, in inexperienced hands, the aspirated supernatant plasma post centrifugation may contain small residual amounts of AC particles, and this may interfere with clot detection, especially for optical clot detection systems.

We hypothesized that for mechanical clot detection instruments, this residual AC would not cause issues in clot detection. Also, we wished to validate the use of DOAC Stop in a liquid concentrate form (DS-L), as this would allow treatment of smaller and more variable plasma sample volumes.

## 2. Materials and Methods

### 2.1. Plasma Samples and DOACs

This is a proof-of-concept study. Pooled normal plasma (PNP) was prepared from selected fresh frozen normal packs kindly donated by the Australian Red Cross Blood Transfusion service. Adsorbed plasma, deficient in the vitamin K-dependent factors, was prepared by adsorbing PNP with 2% of alumina gel (Haematex, Sydney, NSW, Australia). Other factor-depleted plasmas were prepared by adding 5–20% of PNP to individual factor-deficient plasmas (Hyphen BioMed, HBM, Neuville-sur-Oise, France). LA plasma was also from HBM. PNP with 0.3 U/mL heparin was prepared by spiking PNP with unfractionated heparin (Pfizer, Sydney, NSW, Australia).

DOACs, apixaban, rivaroxaban and dabigatran-resembling compounds, were obtained from Chemilieva (Chongquing, China). They were dispersed in dimethyl sulfoxide (DMSO) and further diluted in proprietary stabilizer solutions before addition to the various test plasmas. In most experiments, a representative factor Xa-inhibiting DOAC (rivaroxaban or apixaban) and the thrombin-inhibiting dabigatran were used at concentrations slightly higher than would normally be found in patients on treatment with DOACs.

### 2.2. DOAC Neutralizers/Adsorbents

DOAC Stop Liquid (DS-L; Haematex, Sydney, NSW, Australia) is a 12.5% suspension of DS-AC in a proprietary stabilizer. DS-L functions similarly to DS minitablets, except that it is normally used at 0.01 mL per 0.5 mL of test plasma. This permits neutralization of variable volume plasmas and corresponds to a final concentration of 0.25% DS-AC in plasma. Thus, DOAC Stop Liquid is more user-friendly for small plasma volumes, and this formed part of our investigation.

Alternatively, DS minitablets, each containing 5 mg of DS-AC were typically dispersed in 1.0 mL of a test plasma in a microfuge tube for 5–10 min, then centrifuged at 5000 rpm for 2 min in a microfuge (Eppendorf Model 5424, Hamburg, Germany) and the supernatant removed for testing. For smaller volumes, DS “microtabs” containing half the amount of DS-AC for 0.5 mL samples can be used.

### 2.3. Reagents

APTT reagents used included Triniclot S, PTT-LA (Diagnostica Stago, Asnières-sur-Seine, France), Cephen 5 (lupus anticoagulant resistant, Hyphen BioMed, France), Hemosil Synthasil (IL/Werfen, San Diego, CA, USA), Actin FSL (Siemens, Forchheim, Germany), and Intrinsin LR (Haematex, HTX). All were used with 3 min activation times and 0.025 M calcium chloride for recalcification.

Prothrombin time (PT) reagents included PT Phen (HBM) and Neoptimal (Stago).

Dilute Russell viper venom time (dRVVT) reagents included Staclot DRVV-Screen and Confirm (Stago), Hemoclot LA-S (HBM) and Go-DOAC Test (DCT, HTX). Go DOAC is a dRVV-based clotting reagent modified for higher sensitivity to all DOACs.

All clotting tests were carried out in duplicate in Stago ST4 instruments using 50 µL plasma samples and 100 µL of PT reagents, or 50 µL of dRVVT reagents, or 50 µL APTT reagents followed by 50 µL 0.025 M calcium chloride.

## 3. Results

### 3.1. Investigating the Effect of DS-AC “Contamination”

In the first part of this investigation, we assessed how much DS-AC might be tolerated in clotting tests in our mechanical clot detection instrument. DS-AC was added from either regular DS minitablets (one minitab/0.5 mL) or DS-L (0.02 mL/0.5 mL), thus providing 10 mg/mL (1%) in pooled normal plasma (PNP) initially (this is double the usually recommended concentration of DS). Then, three serial dilutions were carried out in 50 µL volumes of PNP. A similar approach was used in rivaroxaban-spiked PNP (500 ng/mL, R500) and dabigatran-spiked PNP (500 ng/mL, D500). All test plasma samples were gently mixed for 10 min, then used directly (i.e., without centrifuging) in a ST4 mechanical clot detection instrument using the DOAC Test (DCT, Go DOAC™, HTX), and an APTT test using Cephen 5 reagent.

**Figure 1:**
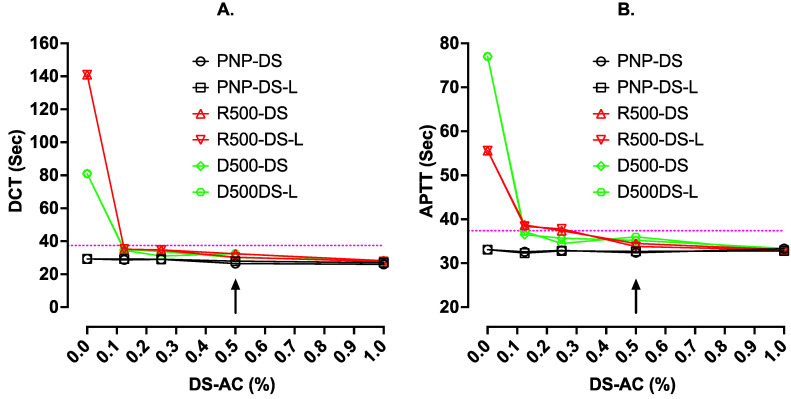
DCT (**A**) and APTT (**B**) test results in normal plasma and plasma spiked with rivaroxaban and dabigatran using DS-AC without centrifugation. The arrow indicates the normal concentration of DS-AC or DS-L used to absorb DOACs, such as rivaroxaban (R500) or dabigatran (D500) as used in this experiment.

The results are shown in Figure 1. This shows that in PNP alone, the presence of up to 1% DS-AC from either minitablets or DS-L had no effect on APTT but yielded slight shortening of the DCT results. In R500 and D500 plasmas, the long test results were shortened back close to normal by higher levels of either adsorbent (i.e., DS-AC or DS-L). There was little difference between results with the two sources of DS-AC. All the subsequent work used DOAC Stop Liquid.

This figure shows the clotting time results (sec) with DCT (Go-DOAC Test) and an APTT test with Cephen 5 reagent on uncentrifuged plasma samples containing varying dilutions of activated charcoal (AC). AC was from either regular DOAC Stop minitablets (Reg. DS) or new liquid DOAC Stop-L (DS-L). Plasma samples were PNP with or without 500 ng/mL of rivaroxaban (R500) or dabigatran (D500). The dotted horizontal lines indicate the upper limit of the normal reference range for the assay, and the arrow indicates the current recommended final concentration of DS at 0.5%. At this concentration, using either regular DS or the new DS-L, clotting times using DCT or APTT normalized to within the normal reference range for both assays.

### 3.2. Effect of Incubation/Mixing Time with DOAC Stop-Liquid

Currently, DS is used for 5–10 min with test plasmas before being centrifuged. We investigated if a shorter mixing time might be possible, with use of the more rapidly dispersing DS-L in test plasmas. We also wanted to check how effective DS might be with higher concentrations of DOACs if using various different testing reagents.

**Figure 2:**
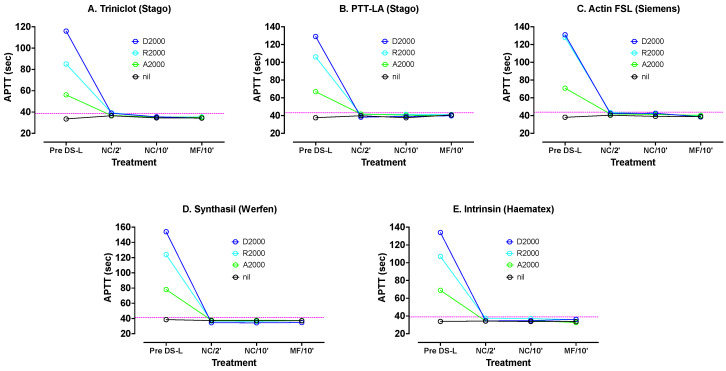
APTT results using various APTT reagents and PNP spiked with apixaban, rivaroxaban and dabigatran before and after treatments with DS-L. (**A**) Triniclot (Stago), (**B**) PTT-LA (Stago), (**C**) Actin FSL (Siemens), (**D**) Sythasil (Werfen), (**E**) Intrinsin (Haematex).

PNP was spiked with dabigatran, apixaban and rivaroxaban, all at 2000 ng/mL. These levels can be considered as well exceeding normal in vivo DOAC concentrations, as we wished to assess how well DS handled such high DOAC samples. These samples were tested with various APTT reagents: Synthasil (IL/Werfen), Triniclot S (Stago), Intrinsin LR (Haematex), PTT-LA (Stago) and Actin FSL (Siemens). The results are shown in Figure 2. All APTT results were significantly prolonged by all three DOACs, but the most by dabigatran and the least by apixaban. Synthasil showed the highest sensitivity and Triniclot the least sensitivity.

DS-L was added to samples of each DOAC plasma and PNP to 0.5% final concentration. APTT tests were carried out directly on samples without centrifuging after 2 and 10 min. Then, each remaining sample was microfuged for 2 min at 5000 rpm and each supernatant plasma again tested with each APTT reagent.

Figure 2 shows APTT results with various reagents using PNP spiked with 2000 ng/mL apixaban, rivaroxaban or dabigatran, before and after treatments with DS-L. The DS-L was mixed with each DOAC plasma for 2 min initially. Sub-samples were removed and tested directly with each APTT reagent without centrifuging (NC/2′). The mixing was continued for another 8 min, and another subsample was tested directly (NC/10′). After the same 10 min mixing the remaining volume was microfuged and the supernatant tested (MF/10′). The dotted horizontal lines indicate the upper limit of the normal reference range for the assay. DS-L led to normalization of all APTT tests under all treatments.

There was good reversal of the DOACs at these high concentrations, even after only 2 min of mixing, although better normalization occurred after 10 min. There was little difference between APTT results on plasmas containing AC after 10 min and plasmas without AC obtained after microfuging. Thus, DS-L was highly effective even with these excessive DOAC levels, and might be able to be used with shorter incubation time than with regular DS, as well as allowing to omit the centrifugation step.

**Figure 3:**
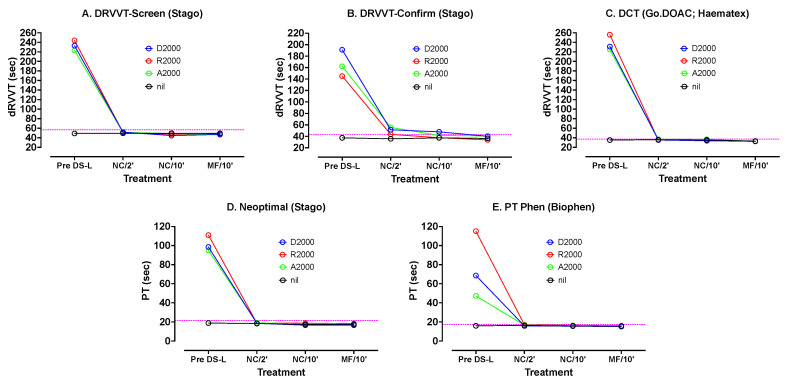
Results of other clotting tests with various reagents on PNP spiked with apixaban, rivaroxaban and dabigatran before and after treatments with DS-L. (**A**) dRVVT Screen (Stago), (**B**) dRVVT Confirm (Stago), (**C**) Go DOAC (Haematex), (**D**) Neooptimal (Stago), (**E**) PT Phen (Biophen).

Similar testing was carried out using other hemostasis tests and reagents (Figure 3). The dRVVT Screen and Confirm reagents from Stago as well as DCT (GoDOAC; Haematex) test results were most prolonged by the DOACs. The PT test results using Neoptimal and PT-Phen were less affected. In most cases there was a rapid correction of the DOAC-prolonged results after only 2 min incubation with DS-L, with a further slower correction back to the result with PNP after 10 min. As with the APTT reagents, there was very little difference between results after 10 min on test samples containing or not containing the DS particles (i.e., centrifugation or no centrifugation).

Figure 3 shows various other tests carried out on PNP samples spiked with 2000 ng/mL DOACs, using the three DOACs and treated exactly as described for Figure 2. The dotted horizontal lines indicate the upper limit of the normal reference range for the assay. DS-L led to normalization of all tests under all treatments.

### 3.3. Tests on Centrifuged vs. Non-Centrifuged DOAC Plasmas

**Figure 4:**
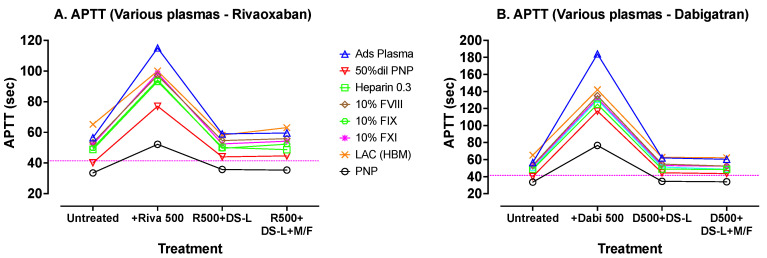
APTT tests carried out with Synthasil on a wide range of test plasmas. Abbreviations: Ads Plasma, absorbed plasma; LAC (HBM), lupus anticoagulant positive control (Hyphen BioMed).

A wider range of abnormal plasmas was then prepared in vitro and tested with the more commonly used Synthasil APTT reagent. This APTT test panel included plasmas with most of the abnormalities detectable using an APTT assay. Each plasma was tested before and after spiking with 500 ng/mL of either dabigatran or rivaroxaban. Then, each DOAC-spiked plasma was mixed with 0.25% DS-AC for 5 min and divided in two. One sample was microfuged for 2 min at 5000 rpm and the supernatant, which was then clear of AC, was tested as usual. The second sample which contained 0.25% AC was added directly to a ST4 cuvette and tested similarly. The results are shown in Figure 4.

Figure 4 shows APTT tests carried out with Synthasil (IL/Werfen) on a wider range of test plasmas. The dotted horizontal lines indicate the upper limit of the normal reference range for the assay. Results on the initial (untreated) plasmas are sequentially followed with results after spiking the plasmas with 500 ng/mL of rivaroxaban (Figure 4A) or dabigatran (Figure 4B), with resultant evident elevations in APTT. Treatment of these DOAC-containing plasmas with DS-L (without centrifugation) showed, in each case, correction of the APTTs to values close to the initial untreated APTT values (except for a slight shortening with the LA plasma). Further processing of the DS-L treated samples by microfugation did not further improve APTT test times.

These results showed the expected APTT prolongations with the abnormal plasmas in comparison with normal APTT in PNP. Results were greatly prolonged by the addition of either DOAC, albeit more so by dabigatran (Figure 4B) than by rivaroxaban (Figure 4A). After treatment with 0.25% activated charcoal from DS-L, the APTTs reverted back to close to their initial untreated values. Similar results were obtained on plasmas after centrifugation to remove the DS-AC, indicating that this additional processing by centrifugation was not required when using this mechanical clot detection system.

**Figure 5:**
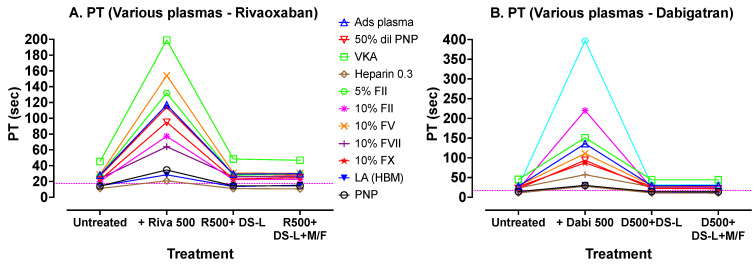
Results of PT tests carried out with Biophen PT Phen reagent on various plasma samples.

A similar set of experiments was performed for PT, including most extrinsic factor deficiencies, and using Biophen PT reagent. The results are shown in Figure 5. These results show a similar pattern to those of APTT, with good correction between the added DOACs and restoration to initial results whether centrifuged and clear of DS-AC, or else still containing the 0.25% AC adsorbent (i.e., no centrifugation).

Figure 5 shows results of PT tests carried out with Biophen PT Phen (Hyphen Biomed) reagent on various plasmas processed in the same sequence as those in Figure 4. The dotted horizontal lines indicate the upper limit of the normal reference range for the assay. Again, addition of DOACs prolonged the PTs compared to untreated samples, and treatment of these DOAC-containing plasmas with DS-L (without centrifugation) showed, in each case, correction of the PTs to values comparable to initial untreated PT values. Further processing of the DS-L treated samples by microfugation did not further improve PT test times.

**Figure 6:**
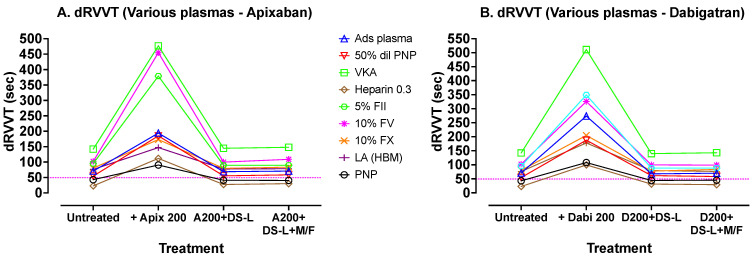
Results of dRVVT tests carried out with Hyphen BioMed Hemoclot LA-S reagent on various plasma samples.

Finally, a similar approach was used to assess dRVVT effects, using Hemoclot LA-S (Hyphen Biomed) dRVVT screening reagent, but using abnormal plasma samples that would give abnormal test results using the dRVVT. The DOACs used in this experiment were apixaban and dabigatran, at lower concentrations (200 ng/mL), due to the higher sensitivity of this test to DOACs. The results are shown in Figure 6. These results show a similar pattern to previous results, with good correction of the prolonged test results using the added DOACs, following addition of DS-L, whether cleared of DS-AC by centrifugation or not.

Figure 6 shows dRVVT tests carried out with Hyphen BioMed Hemoclot LA-S reagent on various plasmas, again processed using the same sequence as those in Figure 4. In this experiment, apixaban (A) was used instead of rivaroxaban. Dabigatran and apixaban were used at lower concentrations here (200 ng/mL) due to their larger inhibition effect on this test. The dotted horizontal lines indicate the upper limit of the normal reference range for the assay. Again, addition of DOACs prolonged the dRVVTs compared to untreated samples, and treatment of these DOAC containing plasmas with DS-L (without centrifugation) showed, in each case, correction of the dRVVTs to values close to the initial untreated dRVVT values. Further processing of the DS-L treated samples by microfugation did not further improve dRVVT test times.

### 3.4. Replicate Testing

We hypothesized that if AC particles did interfere with these clotting tests, that the mechanism may lie with the oscillating rolling ball system within the ST4 cuvettes. Thus, the possible interference of AC on the precision of APTT testing was investigated.

APTT results were not significantly changed by the presence of 0.5% AC. Multiple APTTs with Cephen 5 on PNP and an alumina adsorbed abnormal plasma were carried out. Then, the same tests were carried out with these plasmas spiked with 0.5% AC from DOAC Stop-L (*n* = 6). There was no significant variation in PNP versus PNP containing 0.5% AC, with a clotting time of 34.0 ± 0.13 s and 33.8 ± 0.19 s, respectively. Similar results were observed in adsorbed abnormal plasma with and without 0.5% AC, with clotting times of 72.4 ± 0.37 and 72.4 ± 0.45 s, respectively (mean ± SEM).

## 4. Discussion

DS was originally designed as a simple sample pre-treatment to neutralize DOACs and provide test plasmas free of DOACs after centrifugation to remove the DS adsorbent material. The quickest centrifugation method is with microfuges operating at high speeds. These usual have an angled rotor so that some sediment is deposited along one side of the centrifuge tube. Removal of plasma with a plastic dispenser, especially in inexperienced hands, can dislodge some of the AC particles and there is potential concern that the residual AC may influence clotting tests after DS treatment, in particular with optical detection methods/analyzers.

We hypothesized that this may not be a problem with mechanical clot detection systems. The main manufacturer of these mechanical clot detection instruments is Stago Diagnostics, with instruments using magnetically driven reciprocating stainless steel balls in a cuvette. We did have some concerns that particles of AC might interfere with the frictional resistance involved in the rolling of the balls. Friction might be expected from the AC particles just like small rocks on a road. However, our experiments discounted that such a problem occurs, at least with the reagents and instrument used here, even at the highest levels of activated charcoal used (1% DS-AC), representing twice the recommended amount.

This study also demonstrates a specific advantage of physical (i.e., mechanical) clot sensing devices over those which rely on photo-optical turbidity in terms of DS use. The latter instruments are influenced by a variety of substances that may interfere with clot detection, including the classical hemolysis, lipemia and icteric (i.e., bilirubin), or ‘HIL’; such samples may be routinely excluded from approved testing, unless the instrument has the capacity to adjust to different detection wavelengths less susceptible to HIL interference. Instead, physical clot detection/sensing is not affected by HIL. Similarly, reagents containing turbidity, such as kaolin, are generally no longer accepted for use in some tests, such as those for LA testing, for similar reasons around optical interference; instead, alternate instrumentation not reliant on optical clot detection can routinely use kaolin in tests such as the activated whole blood clotting test. In a similar way, the presence of DS does not appear to adversely affect clotting times using a mechanical clot detection system.

Of interest, DS has recently been used to remove apixaban from whole blood samples, also without centrifugation [26]. Testing in this report was performed using rotational thromboelastometry (ROTEM). Blood samples were simply mixed with a minitablet of DS and tested with a factor Xa-based reagent sensitive to apixaban. The particles of activated charcoal did not interfere with this viscoelastic sensing system, nor with the reagent used.

There is a possibility that some components of a reagent used may be also bound by activated charcoal during the time of testing. It has been shown that DS-AC mainly binds hydrophobic, positively charged compounds with molecular size below approximately 3000 Daltons [27]. Thus, phospholipids (which usually form liposomes of high molecular weight), surface activators, factor Xa and tissue factor are unlikely to be bound.

It is probable that residual AC particles in a plasma might affect the detection of a clotting endpoint, if a turbidimetric method using light scattering were to be used (i.e., optical detection). Photoelectric clot-detecting instruments are used more widely for clotting tests on plasmas than mechanical methods. Alternatively, there are several physical (i.e., mechanical) clot-assessing devices currently available. As previously noted, the main supplier of these for hemostasis laboratories is the Stago line of instruments, starting from the Start ST4 to the Satellite to the STA Max and the StaR evolution (Diagnostica Stago). In addition, there are the current viscoelastography methods including ROTEM (Werfen), ClotPro (Enicor, Chesterfield, UK) and TEG (thromboelastography; Haemonetics Corporation, Boston, United States), and potentially newer emerging instruments [28,29,30,31]. In addition, there are instruments such as the Merlin ball coagulometer (Merlin Medical); these originated from the tilt-tube clotting method and depend on the rapid increase in viscosity at a clotting endpoint being detected electro-magnetically.

Ideally, testing hemostasis in patients would utilize DOAC-free samples. An alternative is to eliminate the unwanted effect of DOACs by using reagents insensitive to these agents. However, no current hemostasis reagent, including any for PT, APTT or dRVVT, contains any in situ DOAC neutralizer. Thus, DOAC neutralization requires exogenous addition of a neutralizer. In theory, this could be achieved with current therapeutic DOAC reversing agents, andexanet alpha (for the anti-Xa DOAC agents) and idarucizumab for dabigatran. However, these may show unwanted side effects on clotting tests when used in vitro [32].

In studies to date, DS has been shown to be an effective DOAC neutralizer for a wide variety of clot-based assays, but especially those used for LA detection [4,9,10,11,12,13,14,15,16,17,18,19,20,21,22]. In our study, we could show that liquid DOAC Stop (DS-L) may offer an even better outcome, given that it is rapidly effective against all types of DOACs, even with very high DOAC concentrations. Moreover, the centrifugation step may be avoided if testing is carried out on a mechanical or impedance clot-detecting instrument, thereby simplifying the procedure and reducing the time required.

As noted in the introduction, DS represents one of the three current commercial DOAC neutralizer options, with DOAC Remove [24] and DOAC Filter [25] representing the other options. All three products have been shown in various studies to effectively remove DOACs ahead of various hemostasis tests [4,9,10,11,12,13,14,15,16,17,18,19,20,21,22,24,25,32,33,34,35,36,37,38,39,40,41,42], although their utility to remove DOACS ahead of LA testing is perhaps most reported [4]. Nevertheless, their utility has also been shown for other hemostasis assays, including thrombophilia testing and global hemostasis assays [9,10,11,12,13,14,15,16,17,18,19,20,21,22,24,25,32,33,34,35,36,37,38,39,40,41,42].

On the other hand, some concerns over their use have been raised. First, it is possible that in any given patient sample, incomplete DOAC neutralization may occur, especially if the DOAC level is very high. To balance this concern, we have shown in the current study, that DS-L was able to effectively neutralize up to 2000 ng/mL of rivaroxaban, apixaban or dabigatran, in assessed assays (Figure 2 and Figure 3). However, as a limitation, we also need to state that we were not able to assess this for every possibly available hemostasis assay. A second concern is that DOAC neutralizers may inadvertently cause some unwanted reduction in factor activities [15,16,25,35,36]. Accordingly, as a second study limitation, we did not assess this in the current study.

## 5. Conclusions

**Figure 7:**
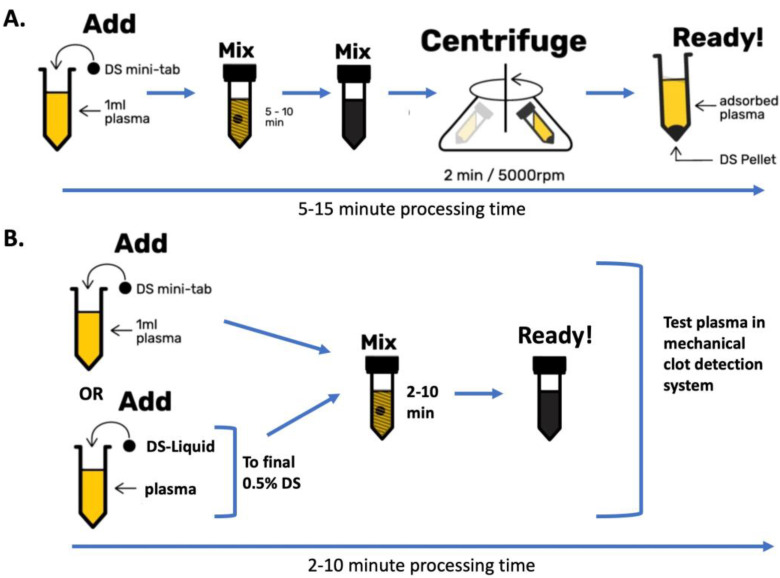
The implications of this study in terms of sample processing. (**A**) Current recommended process. (**B**) Potential options using mechanical clot detection systems.

Residual AC particles from pre-treatment with DOAC Stop appear to have minimal effect on PT, APTT and dRVVT results, at least when carried out with the reagents used here and on a Stago ST4 analyzer. We believe that such findings will be replicated using a wide range of other mechanical clot detection instrumentation, and we look forward to such studies in the future. Indeed, no centrifugation may be required, and clotting tests may be carried out with the full-strength AC present in a plasma sample during the clotting test. DOAC Stop Liquid was shown to perform similarly to current regular DOAC Stop minitablets. A pictorial representation of the new suggested process is shown in Figure 7.

Figure 7A shows the current recommended procedure for use of DOAC Stop (DS); this may require 5–15 min of operator time. This process remains the recommended process if laboratories are using optical detection methods, although the DS minitablet can be replaced with DOAC Stop Liquid (DS-L). Figure 7B shows how the process can be modified for use of test systems using mechanical clot detection. Our study has validated this process for a large number of hemostasis tests performed on the ST4 coagulation analyzer. We await further studies validating this process for other mechanical clot systems, including other Stago analyzers, as well as ROTEM, Proclot and TEG instruments.

## Data Availability

All derived data are shown in manuscript.
